# Origin of “Humbug”

**Published:** 1885-06

**Authors:** 


### Origin of the Word "Humbug."

It may not be much of an honor to note that the word "humbug" is of American origin, yet such being the case, we beg leave to relate how it came into our vocabulary.

Anterior to the dictionary of Noah Webster the word cannot be found. It is there chronicled as a "low word," and is defined to mean "imposition." Now-a-days, the word has become classical, and is now used in polished society, by some of the leading writers, in American Congress, and in the British Parliament. We do not remember of ever having seen the origin of the word explained, and therefore think our readers will be interested in reading the following:

In the summer of 1807, a singular individual, by the name of Homberg, came to Philadelphia, and announced that he would cure all diseases without fee or reward. The "doctor" soon had plenty of patients. He resided at the old White Swan Tavern, in Race street.

Hundreds of people there collected every afternoon, between the hours of four o'clock and dark, and often so dense was the motley crowd as to blockade the street. In the height of this man's celebrity, the late Prof. B. S. Barton, in Chestnut street, declared that this man was actually insane. This was doubted considerably. The "doctor" had his office in the bar-room, where he was caged in a kind of a box about five feet square, partitioned off from the floor to the ceiling on the outside of the room The only aperture to this sanctum, besides a small door through which the oracle entered, and which he carefully bolted inside, was a circular hole six or eight inches in diameter. Through this opening the mysterious man received empty, and passed out with the utmost celerity full vials, each containing six or eight ounces. The "doctor" seemed to understand the diseases of his patients intuitively, as he would continually supply them with medicine without appearing to investigate their cases. He seldom conversed with anybody except in monosyllables, and from his patients he never would hear long stories. All he wanted to know from them was the name of their respective diseases, and they were immediately supplied with medicine. He seldom left his chamber except at his prescribing hours, or in the afternoon, and at daylight in the morning to collect herbs. He was very gruff and reserved in his manner. Upon being questioned about the idea of *gratis* work, he replied, with some hesitancy, "I am not *allowed* to *charge*, but people may *give* anything they please." This was like "method at least in madness," for his apparent indifference to money was the result of shrewd deliberation, and proved as profitable, upon the whole, as the usual practice of making regular charges.

He said he was a German, and said that the reason that he came to America was because "Jesus Christ sent him here." Upon investigation it was found that the kind of medicine he used was "buttercup," which he directed sometimes to be externally applied instead of a Spanish fly-plaster, also common weeds, found in any old field, cut up fine and boiled thoroughly in a large copper kettle filled with water and then strained. This was all emptied into a tub, and concealed under a shelf in his private sanctum, just below the round hole, through which he transacted his business. From this same tub all diseases ranging from heart disease to the simple toothache were treated, and nearly all his patients declared that he was performing wonderful cures.

All at once—as if by a bomb-shell—the secret burst out, and just as quickly the patients *vanished*, as if touched by a magician's wand, leaving the "doctor" all alone in his career. Then came the taunts of the people!

"Were you Homberg-ed?" they would ask each other, and, like the *pebble* which becomes smooth by attrition, the term soon became changed to the somewhat more euphoneous sound of "Were you Humbugged?" "*He* was humbugged!" "*You* have been humbugged!" with the occasional retort, "And so were *you!*" Such expressions were "as familiar as household gods" for months afterwards.

Thus was an event which produced keen mortification at the time, afforded a graphic instance of a "humbug," the term itself originated, and is a convenient addition to our vocabulary.—*Agents' Herald, March*, 1885.

